# Metal–Support Interactions in Heterogeneous Catalysis: DFT Calculations on the Interaction of Copper Nanoparticles with Magnesium Oxide

**DOI:** 10.1021/acsomega.3c00502

**Published:** 2023-03-07

**Authors:** Amir H. Hakimioun, Bart D. Vandegehuchte, Daniel Curulla-Ferre, Kamila Kaźmierczak, Philipp N. Plessow, Felix Studt

**Affiliations:** † Institute of Catalysis Research and Technology, Karlsruhe Institute of Technology, Hermann-von-Helmholtz Platz 1, 76344 Eggenstein-Leopoldshafen, Germany; ‡ Institute for Chemical Technology and Polymer Chemistry, Karlsruhe Institute of Technology, Engesserstrasse 18, 76131 Karlsruhe, Germany; § TotalEnergies OneTech Belgium, B-7181 Seneffe, Belgium

## Abstract

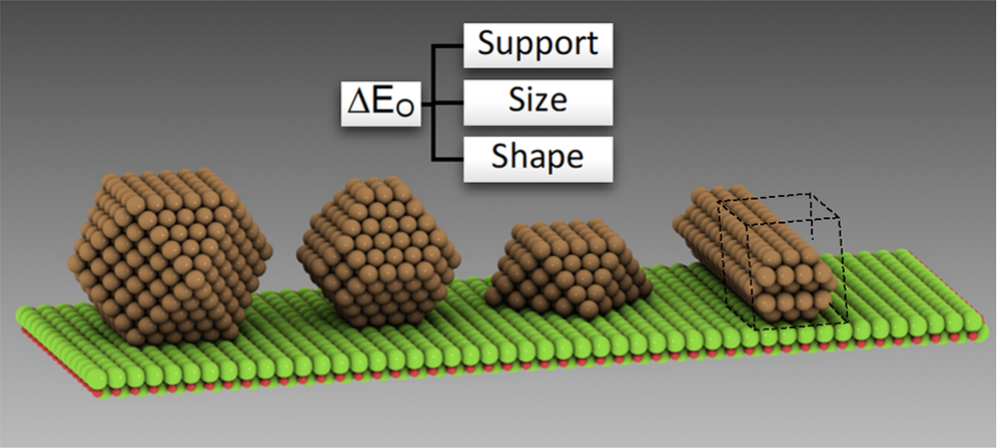

Oxide supports play an important role in enhancing the catalytic properties of transition metal nanoparticles in heterogeneous catalysis. How extensively interactions between the oxide support and the nanoparticles impact the electronic structure as well as the surface properties of the nanoparticles is hence of high interest. In this study, the influence of a magnesium oxide support on the properties of copper nanoparticles with different size, shape, and adsorption sites is investigated using density functional theory (DFT) calculations. By proposing simple models to reduce the cost of the calculations while maintaining the accuracy of the results, we show using the nonreducible oxide support MgO as an example that there is no significant influence of the MgO support on the electronic structure of the copper nanoparticles, with the exception of adsorption directly at the Cu–MgO interface. We also propose a simplified methodology that allows us to reduce the cost of the calculations, while the accuracy of the results is maintained. We demonstrate in addition that the Cu nanowire model corresponds well to the nanoparticle model, which reduces the computational cost even further.

## Introduction

Oxide supported transition metal particles constitute an important class of heterogeneous catalysts, being employed for reactions ranging from hydrogenation over oxidation to emission control.^1^ The composition of such catalysts in terms of oxide and transition metal, greatly determines the interaction between metal particles and the support surface. This interaction additionally both depends on and dictates particle size and shape as well as the long-term stability of the catalysts,^2−10^ with typical sizes of the transition metal particles used in industry being in the nanometer range, mostly somewhere in between 3–20 nm.^11^

Importantly, the activity and selectivity of a catalyst often depend crucially on the exact nature of the particle–oxide interaction.^12−23^ There are multiple ways being discussed via which the oxide support can alter the catalytic function of the particle: indirectly, through changing the shape and thus faceting of the nanoparticle^6,24^ or through an electronic interaction between the support and the particle, changing the electronic structure and thus the d-band of the metal,^4,25−28^ or directly, when the reaction occurs at the metal–support interface with the reactants and intermediates binding to both the transition metal and oxide support.^29,30^ While these phenomena offer a plethora of possibilities to enhance the performance of catalysts,^16^ it also makes the identification of active sites and reaction mechanisms, and thus a knowledge-based improvement, difficult to achieve.

Theoretical studies, mainly based on density functional theory (DFT) calculations, have found widespread application in the catalysis field as they are able to reveal the nature of the interaction of the transition metal with the reactants and intermediates. Furthermore, these calculations are now routinely used to compute transition state energies that can be directly linked to a catalyst’s activity and selectivity.^31−34^ As the size of the real catalytic system is intractable for computations, these are typically performed using simple models of the active site. While small nanoparticles are subjected to quantum size effects,^35−37^ traditional metal nanoparticles (NPs) with diameters larger than 3 nm are conveniently modeled using the extended surfaces of the facets constituting the particle, thus greatly reducing the size of the system. These models do, however, not include support effects other than those on particle shape. To include also other support effects, more and more attempts are made for modeling of supported nanoparticles (NPs).^38−40^ Due to the increasing computational demand with increasing particle size, these models typically consist of particles well below 3 nm, where they are subject to quantum size effects and might thus not be representative of the much larger catalytic system.

An elegant way to circumvent these limitations has been found in so-called “nanowire” or “nanorod” models of the transition metal supported on the oxide of interest.^41−43^ Such an approach ensures that the model mimics the behavior of large particles and is able to simulate the influence of the support while being computationally feasible. The accuracy of these models in representing the true interaction of large particles with oxide surfaces and the metal–support interaction, however, has not been fundamentally addressed to date.

Herein, we study the influence of MgO on copper, measured by changes in the binding strength of an oxygen atom, to evaluate the impact of parameters such as particle size and shape and, most importantly, the effect of the interface. Using supported copper NPs with diameters up to 2.6 nm, we will also compare their performance with nanowire models and make an assessment of how well they can serve as models for metal–support interfaces.

## Methods

All periodic DFT calculations were carried out using the Vienna Ab initio Simulation Package (VASP)^44−47^ version 5.4.1 and the functionalities of the Atomic Simulation Environment (ASE)^48^ python library, employing the Bayesian error functional with van der Waals corrections (BEEF-vdW)^49^ and the projector-augmented-wave (PAW)^46,50^ method with standard potentials for metal atoms and soft PAW potentials for oxygen atoms. Γ-centered *k*-point sampling was used in all calculations. Copper nanoparticles were computed at the Γ-point. For the copper nanowire model, a 2 × 1 × 1 *k*-point was employed. The plane-wave kinetic energy cutoff was set to 350 eV for the calculations of the oxygen adsorption energy on systems including Cu NPs and NW. A 16 × 16 × 16 *k*-point sampling and a cutoff energy of 800 eV were used for the calculations of bulk Cu and MgO. All calculations used a Gaussian smearing with a width of 0.1 eV. The bulk lattice parameters were calculated to be 3.664 and 4.262 Å for Cu and MgO, respectively, in good agreement with experimentally and theoretically reported values.^51−55^ During the optimization of the MgO-supported Cu NPs and NW structures, the positions of Mg and O atoms were fixed (with respect to its lattice in the bulk), whereas the positions of all copper atoms were allowed to move until the forces of relaxed atoms converged to below 0.01 eV/Å. The distance between the copper nanoparticles and the MgO support, which was used for the fixed-geometry models, was obtained from the relaxation of a three-layered copper slab with a unit cell of (1 × 1) on a two-layered MgO slab with the same unit cell size.

The adsorption of oxygen atom was modeled on a p(4 × 4) Cu(111) slab to determine the reference oxygen chemisorption energy and the distance of the atom from the Cu atoms in fcc and hcp positions (used further for the adsorption on the fixed-geometry nanoparticles). The O was allowed to fully relax, while the Cu was kept frozen. The oxygen adsorption energy was calculated as follows
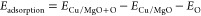
1where *E*_Cu/MgO+O_ stands for the total energy of an oxygen atom adsorbed on a MgO-supported copper nanoparticle, *E*_Cu/MgO_ for the total energy of a MgO-supported copper nanoparticle, and *E*_O_ for the energy of an oxygen atom (1/2 *E*_O2_).

As shown in the SI (Figure S3), to find a sufficient distance between the periodic images of MgO-supported Cu NPs that guarantees negligible interaction, test calculations of oxygen adsorption energies were performed on some of the Cu NPs by varying the distance between the clusters. The results show that a small distance of 5 Å is sufficient, and for computational efficiency, this separation was chosen. The possible interactions between the periodic images of Cu/MgO structures orthogonal to the surface were avoided by applying a vacuum of ∼16 Å in the *z*-direction.

The calculations of the oxygen chemisorption on different copper FCC-type surfaces (Cu(111), Cu(100), Cu(211), Cu(110), and Cu(321)) were performed using four layers of a Cu slab, where the two bottom layers were fixed to the lattice of copper in the bulk. The copper atoms in the unconstrained layers were relaxed until their forces were smaller than 0.01 eV/Å. For the Cu(111) slab: a unit cell of (3 × 3) with the *k*-point grid of 4 × 4 × 1; for the Cu(100) slab: a unit cell of (4 × 4) with the *k*-point grid of 3 × 3 × 1; for the Cu(211) slab: a unit cell of (3 × 3) with the *k*-point grid of 5 × 4 × 1; for the Cu(110) slab: a unit cell of (2 × 2) with the *k*-point grid of 4 × 6 × 1; and for the Cu(321) slab: a unit cell of (3 × 3) with the *k*-point grid of 3 × 4 × 1 were used. For all of the surfaces, a cutoff energy of 450 eV was used. The oxygen adsorption energy was calculated relative to the energy of 1/2 O_2_ in the gas phase. For visualizing the structures shown, iRASPA^56^ and VESTA^57^ software were used.

## Results and Discussion

The choice of the copper supported on magnesium oxide (Cu/MgO) system is motivated by related studies in methanol synthesis.^29^ The fact that MgO is a nonreducible support and that both MgO(100) and Cu(100) have square unit cells greatly simplifies the Cu/MgO model construction. Our investigations span from cluster sizes of 55 atoms (Cu_55_) to particles consisting of 561 atoms (Cu_561_) that are ∼2.6 nm in diameter. These large particles require a tremendous amount of computational capacity in DFT simulations, and we therefore turn to approximate models using single-point energy calculations, greatly reducing this effort. We chose the oxygen binding energy as this is a simple enough descriptor that is sensitive to the underlying electronic structure of copper^58^ and is often used to estimate the activity of copper as well as other transition metals for the hydrogenation of CO_2_ to methanol.^59^

**Figure 1:**
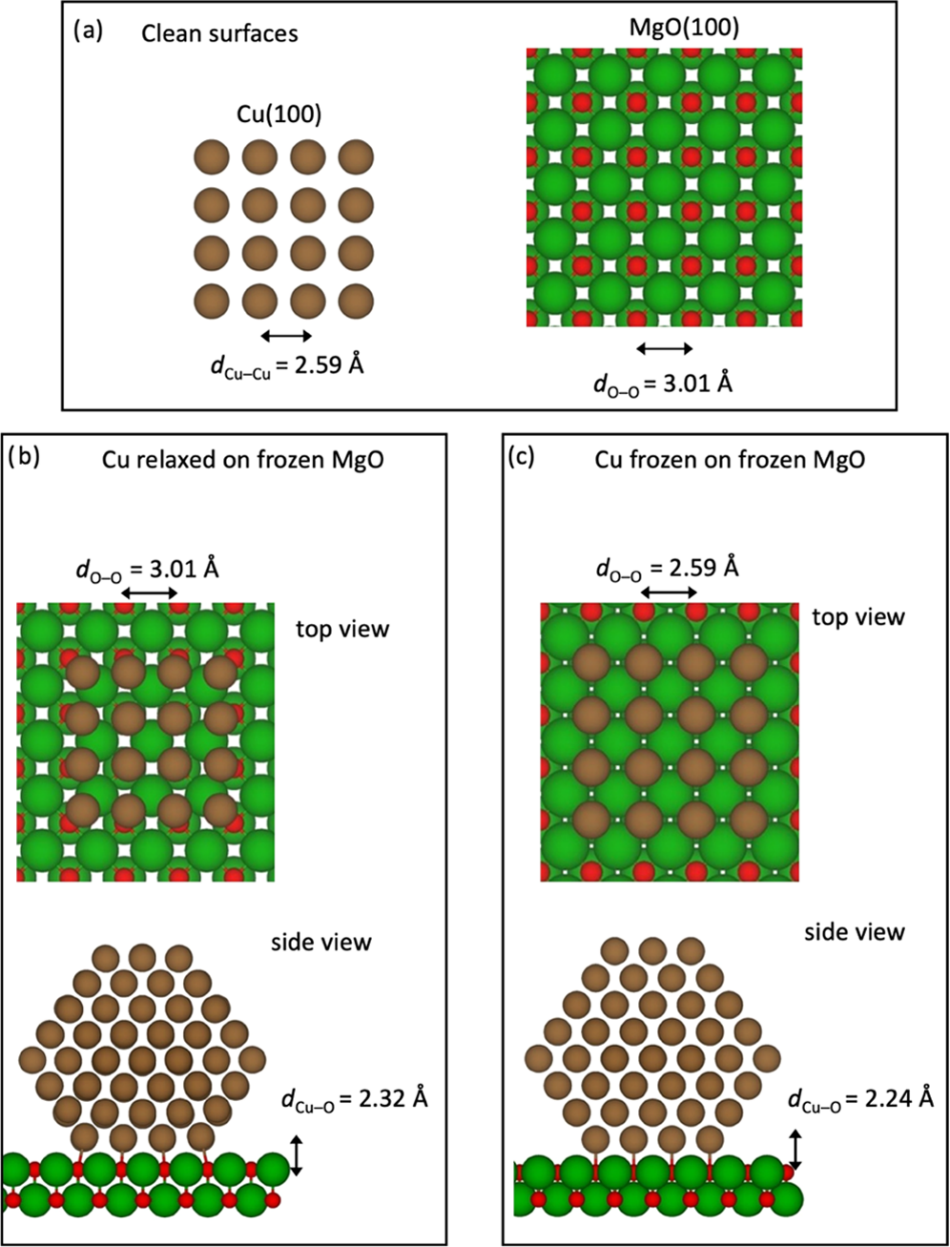
Illustration of the interface models studied in this work (a) Clean surfaces and (b, c) interfaces that arise when using (b) a relaxed Cu_192_ particle on MgO and (c) a Cu_192_ particle with atoms fixed in their bulk positions on top of MgO, where the atomic bulk positions have been scaled to match the lattice of Cu. For the top views, only one layer of Cu(100) is shown; in the case of interfaces, it is the interfacial layer.

First, we start with the influence of magnesia on the intrinsic stability of the copper nanoparticles. The nanoparticles binding with their {100} facet to the MgO(100) show stronger adhesion energies than the others which bind via {111} facets. This is caused by the difference in symmetries and alignments between the two interacting interfaces. This can be observed in Figure 1, which shows the structures of both Cu(100) and MgO(100). For a perfect match at the interface, an interfacial Cu atom would always be placed exactly on top of an oxygen atom. However, due to the fact that the MgO lattice constant is 16% larger (lattice mismatch), this will be increasingly difficult for larger clusters.

Two types of models are considered in this work, First of all, Cu clusters are fully relaxed on a two-layered MgO(100) slab, with the Mg and O atoms frozen in their bulk positions (see Figure 1b). Since this is computationally very demanding, we furthermore explored a simplified model, where the Cu particles are also frozen with the atoms in their bulk positions. Due to the mentioned lattice mismatch, this simplified approach requires one lattice constant to be adjusted and we chose to scale the MgO lattice so that it matches that of Cu and keep Mg and O atoms again fixed in their (scaled) bulk positions (see Figure 1c). The distance of the copper nanoparticles from the support was obtained from a calculation of a Cu(100) slab on MgO(100). This approach was chosen mainly to have strain-free Cu clusters for the subsequent calculation of adsorption energies.

The adhesion energy per copper surface atom that interacts with the MgO support (*E*_adhesion_) is calculated by subtracting the energy of the free-standing Cu NPs (*E*_Cu_) and that of the MgO slab (*E*_MgO_) from the energy of the supported Cu NPs (*E*_Cu/MgO_) normalized through division by the number of copper atoms facing the oxide interface (*n*).
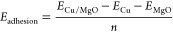
2

**Figure 2:**
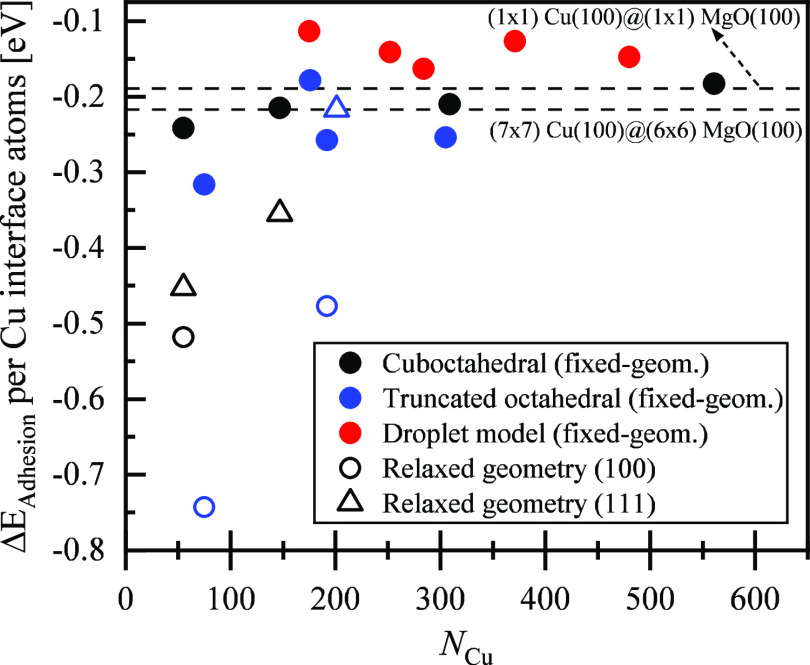
Adhesion energy of copper NPs supported by MgO per interacting surface copper atom plotted against the total number of copper atoms in the nanoparticles. Solid and hollow shapes represent the fixed-geometry and fully relaxed Cu NPs on MgO, respectively. Circle shapes are used to show the adhesion energies of copper NPs on the MgO via their Cu(100) facets, and triangle shapes are used to show the adhesion of Cu NPs on the MgO surface via their Cu(111) facets. Black, blue, and red colors are used to differentiate the series of cuboctahedral (Cu_55_, Cu_147_, Cu_309_, and Cu_561_), truncated octahedral (Cu_75_, Cu_176_, Cu_192_, and Cu_305_), and droplet-like (Cu_175_, Cu_252_, Cu_284_, Cu_371_, and Cu_480_) nanoparticles, respectively.

The results of the adhesion energies of Cu NPs are depicted in Figure 2. The solid circles and hollow shapes shown in Figure 2 represent the adhesion energies of the fixed geometries (Cu bulk lattice constant, see Figure 1c) of copper nanoparticles adsorbing on lattice-matched MgO (compressed by 16% to match the lattice of Cu in the bulk) and relaxed Cu nanoparticles on MgO, respectively (see Figure 1b). The dashed line in Figure 2 at −0.21 eV shows the adhesion energy of a relaxed three-layered (7 × 7) unit cell of a Cu(100) slab on a two-layered (6 × 6) unit cell of a MgO(100) slab (constrained to the MgO lattice in the bulk), and the other dashed line, at −0.46 eV, shows the adhesion energy of a relaxed three-layered (1 × 1) unit cell of a Cu(100) slab adsorbed on a two-layered (1 × 1) unit cell of MgO(100) slab (compressed lattice to match with the copper lattice in the bulk). Theoretical investigations for alumina and silica^60^ showed that adhesion energies computed with either the lattice constant of the metal or that of the oxide adjusted lead to adhesion energies that differ by less than 15 meV/Å^2^, if the symmetry of the metal is not broken and if the strain is <3%. However, as discussed above for the case considered here, the small supercell with MgO compressed by 16% is shown to give adhesion energies that agree well with the larger supercell with <1% strain. The reason is that strain-free supercells lead to incommensurate interfaces, while the compressed MgO(100) is less reactive despite forming a commensurate interface with Cu(100), as discussed below in more detail.

Relaxed clusters binding to MgO(100) via the Cu(100) facet show the strongest adhesion energy per interfacial Cu atom (−0.5 to −0.75 eV). We attribute this to the fact that the very small fcc(100) facets can more easily adapt to the lattice mismatch. For larger interfaces, as described by the (7 × 7)-Cu(100) on (6 × 6)-MgO(100) model, the surfaces are not atomically aligned, leading to much weaker binding, on the order of −0.2 eV per atom. The structural models based on the MgO(100) support adjusted to the lattice constant of Cu(100) show a comparably weak adhesion energy. This applies to the periodic interface of (1 × 1)-Cu(100) on (1 × 1)-MgO(100) and the models using frozen Cu clusters (solid circles).

**Figure 3:**
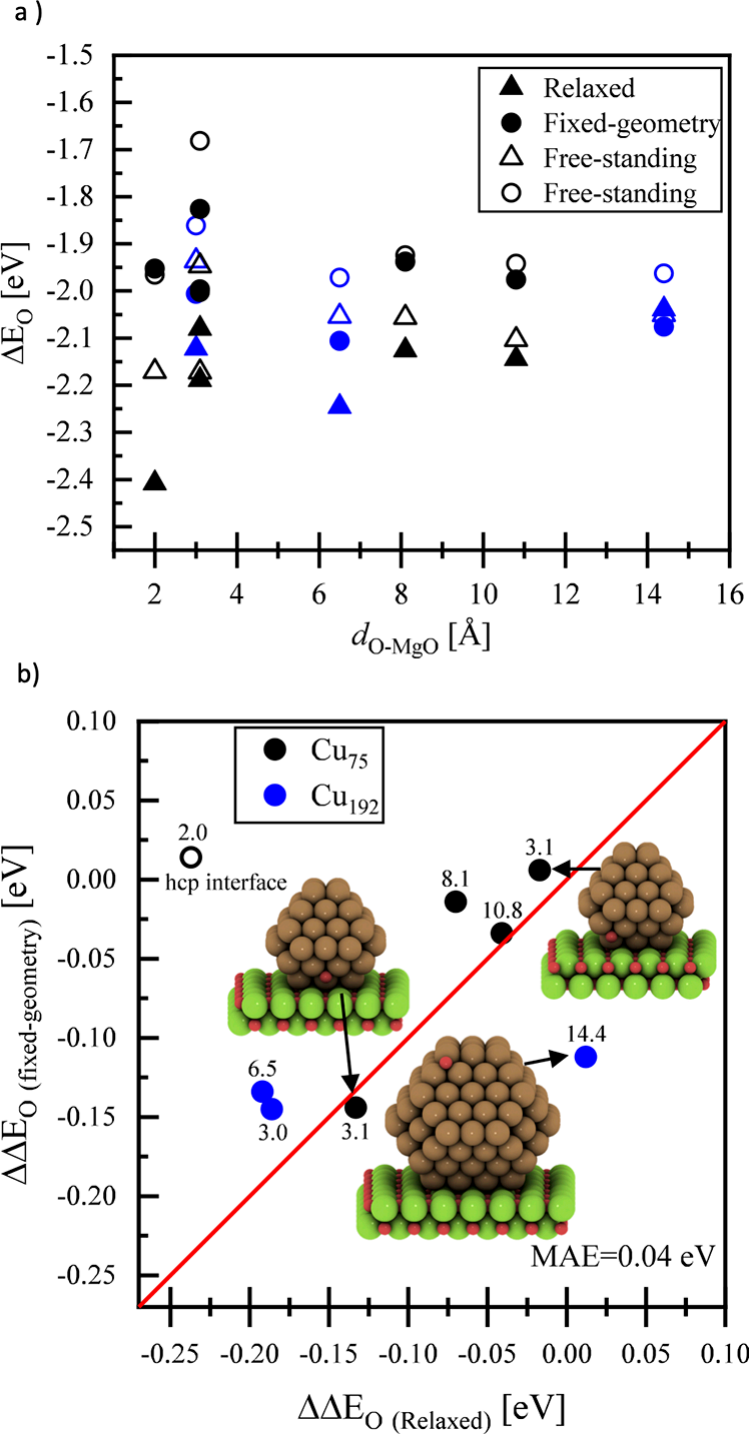
(a) Calculated adsorption energy of oxygen atoms (relative to 1/2 gas phase O_2_) on Cu_75_ (black symbols) and Cu_192_ (blue symbols) both supported by magnesia (full symbols) and free-standing (hollow symbols) against the distance of the adsorbate from the support. The triangle and circle points represent the optimized and fixed-geometry structures, respectively. (b) Parity plot of the difference of the oxygen adsorption energy between supported and free-standing fixed geometries of Cu_75_ and Cu_192_ against the same calculated energies of relaxed structures. The numbers on each of the points in the figure show the distance of the oxygen atom adsorbate from the magnesia support.

Due to the strong interaction of truncated octahedral copper nanoparticles with MgO, we also used them to introduce and validate our simplified methodology against full geometry optimized systems. To this end, the oxygen adsorption energy on various adsorption positions on Cu_75_ and Cu_192_ truncated octahedral particles was calculated. These results are shown in Figure 3a. The oxygen adsorption energies were calculated from full relaxation and single-point calculations of MgO-supported copper nanoparticles and the adsorbate (oxygen atom). For single-point calculations, the distance of the adsorbate from the adsorption position of the copper nanoparticles was taken from the relaxation of oxygen atoms adsorbed on FCC and HCP positions on a frozen four-layered Cu(111) slab, where all Cu atoms were fixed at their bulk positions. In all cases, the oxygen atom was adsorbed on FCC(111) facets of the Cu NPs.

The differences of the oxygen adsorption energies between the supported and unsupported (free-standing) fixed geometries of Cu_75_ and Cu_192_ nanoparticles are plotted against the same differences from the fully relaxed particles in Figure 3b.

As can be seen from Figure 3b, the computationally cheaper procedure of calculating single-point energies yields comparable results with those from full relaxation, with a mean absolute error below 0.1 eV. The only exception is the oxygen adsorbed directly at the interface between the copper particle and the MgO support (hollow circle in Figure 3b). Since there is a significant interaction of the oxygen with both the copper particle and the MgO surface, only fully relaxed particles yield reasonable results.

Having verified that the static model employing single-point energy calculations is in fact able to reproduce the results and trends from the full geometry optimized particles, we now turn to calculating the oxygen adsorption on various copper clusters and particle models on the MgO(100) surface.

**Figure 4:**
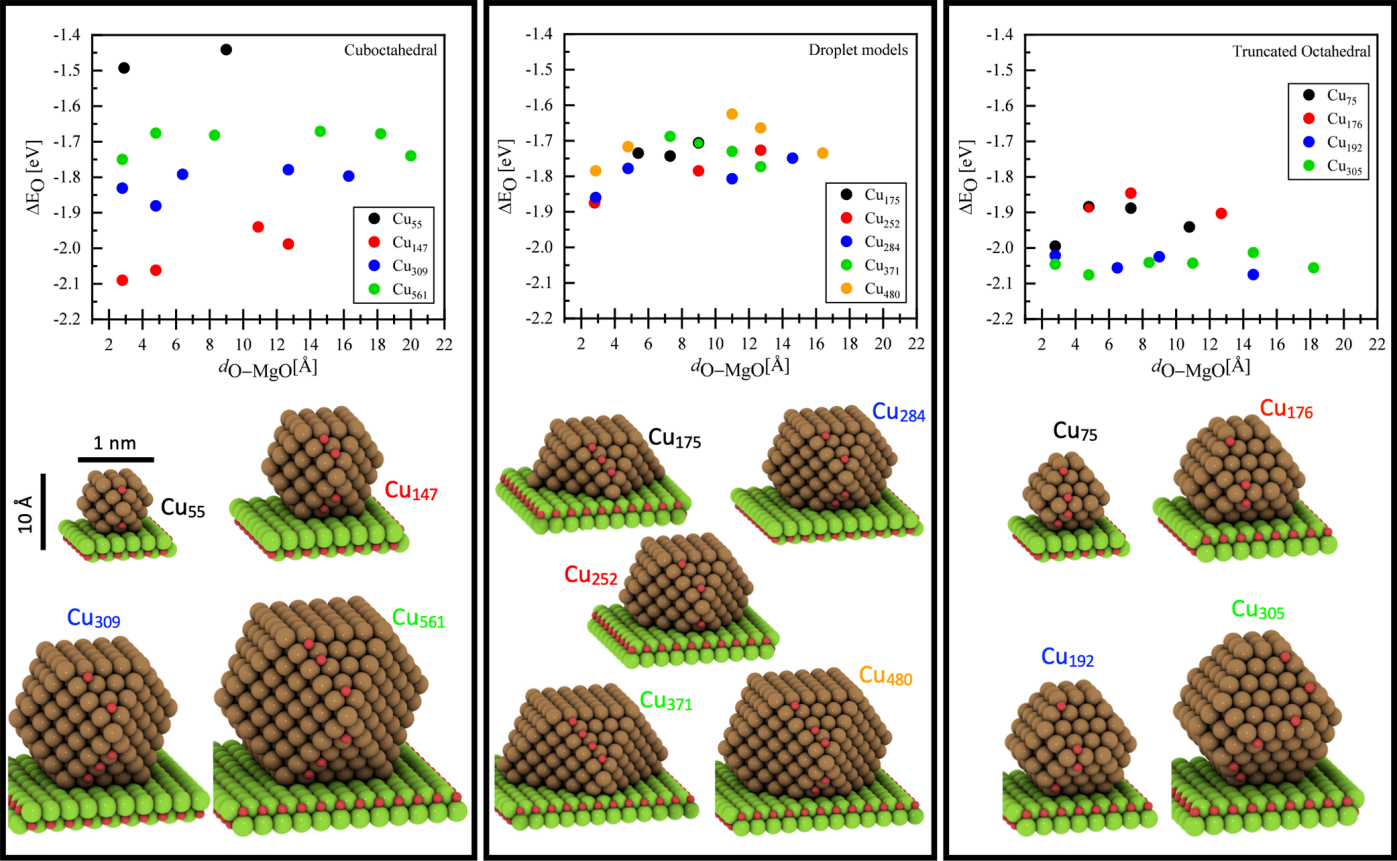
Oxygen adsorption energies calculated on the fixed structures of the MgO-supported copper NPs. The lattice constant of MgO, as described in the model section, is compressed to match with the lattice constant of copper in the bulk (Cu–Cu distance of ∼2.59 Å). From left to right: oxygen adsorption energy on cuboctahedral, droplet-like models, and truncated octahedral copper nanoparticles against the distance of oxygen (adsorbate) from the oxide interface.

The extent of interaction of the MgO(100) surface with the various copper clusters and particles on the corresponding oxygen binding energy is shown in Figure 4 for a series of truncated octahedral (Cu_75_, Cu_176_, Cu_192_, and Cu_305_), cuboctahedral (Cu_55_, Cu_147_, Cu_309_, and Cu_561_) and droplet models (Cu_175_, Cu_252_,Cu_284_,Cu_371_, and Cu_480_).

The impact of oxygen adsorption site distance from the support was evaluated, only considering positions on the copper clusters that are equivalent in terms of adsorption site geometry (see structures and adsorption sites in Figure 4). As can be seen from Figure 4, the influence of the MgO support on the oxygen binding energy is rather small (<0.10 eV for most cases). There is a slight, albeit not very pronounced, effect at a close distance to the MgO(100) facet, where oxygen is binding to a position on the copper clusters less than about 4 Å from the MgO(100) plane. Here, the oxygen adsorption energy seems to slightly increase for some clusters by up to −0.17 eV. Importantly, the MgO support has only a small effect on the oxygen binding energy for all clusters and particles considered in this study, independent of their size and shape. The influence of the MgO support (see Figure S4), for some of the nanoparticles, is given as the difference between the oxygen chemisorption energy of the unsupported (free-standing) copper clusters and their MgO-supported counterparts (ΔΔ*E*_O_) as a function of the vertical distance of the chemisorbed oxygen from the MgO(100) plane. As can be seen, large variations only exist when comparing NPs of different sizes rather than one size as a function of the metal–support interaction. The size effect is similar to what has been observed in a recent study.^36,37,58,61^

**Figure 5:**
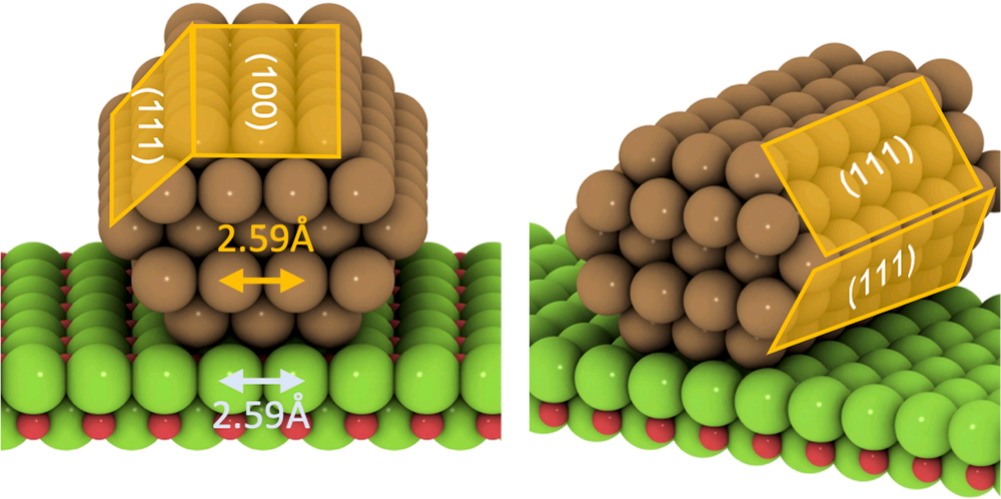
Left: Front view and right: perspective view of the Cu(7 × 5 × 5)/MgO nanowire model. The Cu(111) and Cu(100) planes are shown in the figures. Colors: Cu (brown), Mg (green), and O (red).

Due to the high computational cost of DFT calculations for large systems, simplified computational models of the particle–support interface have been introduced recently. These are based on nanowires (NWs) of the transition metal interacting with the support.^41^ By using nanowires, one can achieve a model with a smaller number of atoms per unit cell that mimics the electronic structure of larger particles as a metallic character is obtained due to the periodic calculation. One therefore avoids the quantum size effects of sub-nanometer clusters that are typically used in computational studies. Here we employ a Cu nanowire model to simplify the calculations of the larger nanoparticles. The MgO-supported copper nanowire used in this study is shown in Figure 5. Similar to the models of nanoparticles (shown in Figure 4), the structures of the Cu NW as well as MgO were also fixed to the bulk lattice of copper to perform single-point calculations on the systems generated. The surface of the support is modeled by two layers of MgO(100) with a p(7 × 6) unit cell. The distance between two nanowires is approximately 5 Å, which we verified as sufficient to suppress possible interactions between the periodic images (see Figure S3).

Having shown that the support effect is visible only at the exact Cu–MgO interface, we have evaluated the influence of the adsorption site for all other adsorption positions on all considered models more generally using the concept of generalized coordination numbers (GCN), introduced by Sautet and co-workers.^62^ GCNs are calculated by counting the nearest neighbors of the atoms on which adsorption takes place (*n_j_*), weighing them by their own coordination numbers (cn(*j*)) and dividing by the maximum number of neighbors for a given adsorption position (cn_max_).
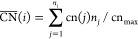
3

**Figure 6:**
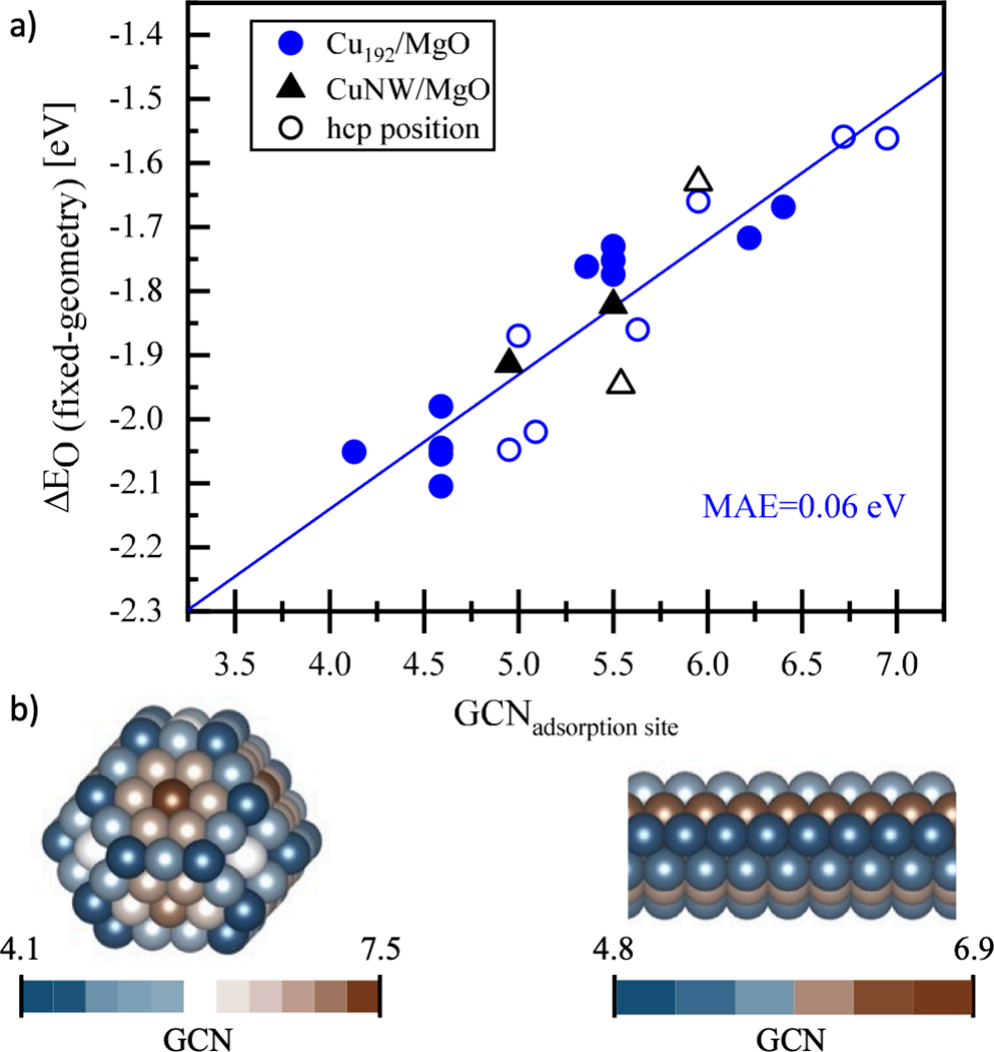
(a) Oxygen adsorption energies calculated on different positions of MgO-supported Cu_192_(blue circles) and Cu nanowire (black triangles) fixed-geometry against the calculated values of GCN of the adsorbing positions. The blue fitted line shows the extrapolation of the oxygen adsorption values calculated for Cu_192_/MgO. The hollow points in the figure represent the adsorption on HCP positions on the structures. (b) Left: The structure of a truncated octahedral Cu_192_ nanoparticle. Right: The side view of the structure of a Cu(7x5x5) nanowire. The atoms are colored with respect to their GCN values for which the range of the values is shown by the color scale bars below the structures. We observe the general trend that an increasing adsorption strength correlates with a decreasing GCN.

This analysis reveals how the variation of the adsorption site on the cluster and particle models is affecting the oxygen adsorption strength. Figure 6 shows the adsorption energies of oxygen (Δ*E*_O_) for various positions on both the MgO-supported Cu_192_ and Cu(7 × 5 × 5) nanowire structures as a function of the GCN of the respective adsorption positions. As evident from Figure 6, Δ*E*_O_ is mostly a function of GCN, and the relation has a low mean absolute error (MAE).

**Figure 7:**
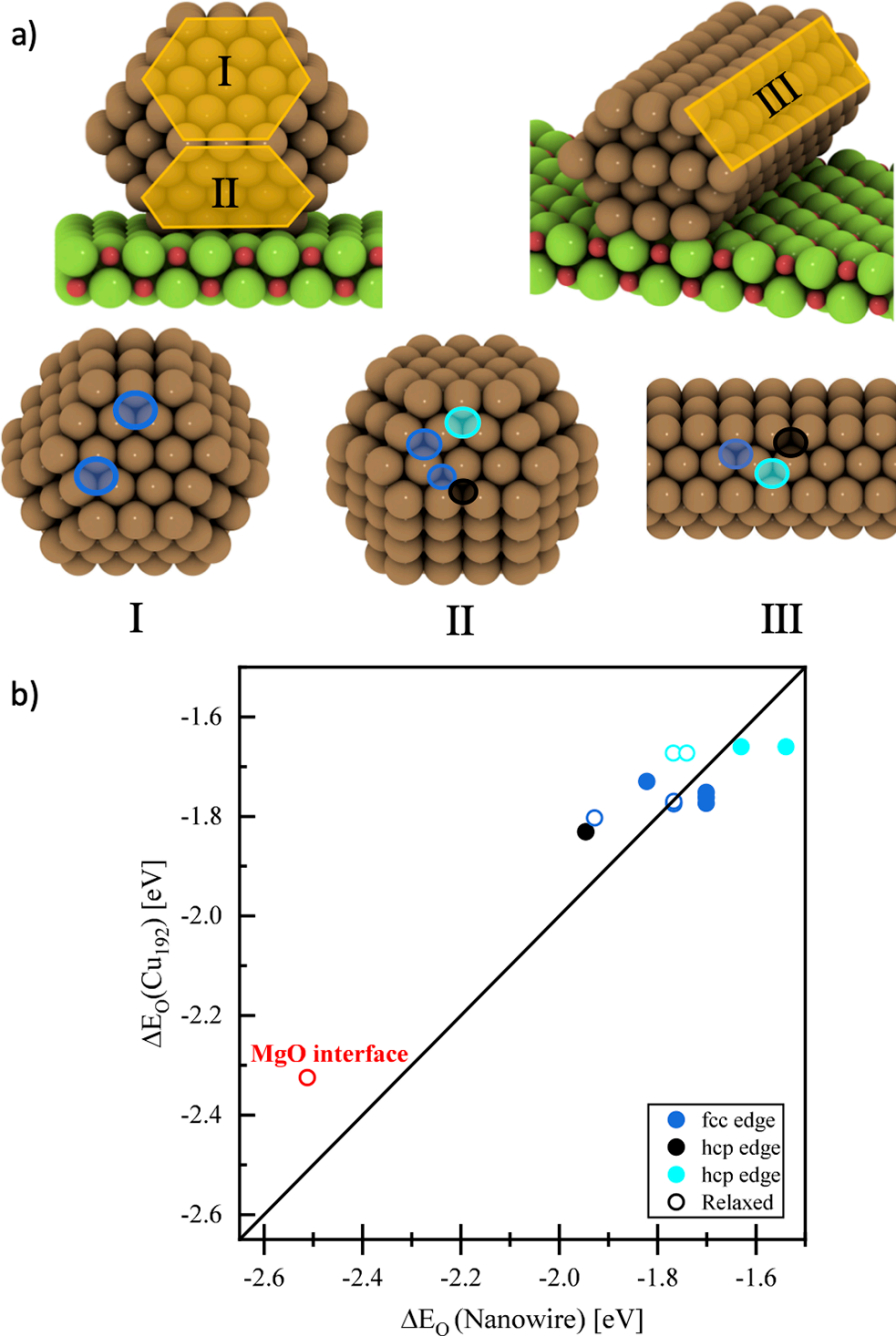
(a) Left: The structure of the 45° rotated Cu_192_ NP on MgO. Right: The 45° rotated Cu(7 × 5 × 5) NW on MgO. The numbered (I, II, and III) structures are {111} facets on the upper and lower planes of Cu_192_ and the upper plane of Cu_NW_, respectively. The blue, black, and cyan highlighted circles depicted on the adsorption sites of the structures represent the intersecting FCC position between {111} and {100}, HCP position between {111} and {111}, and HCP position between {111} and {100} facets, respectively. (b) Comparison of calculated oxygen adsorption energies on different adsorption sites between Cu_192_/MgO and NW/MgO. The solid and hollow circles represent the energies of fixed-geometry structures and fully relaxed ones, respectively.

Next, we compare the MgO-supported NW model with calculations of Cu_192_/MgO (Figure 7). Figure 7a shows the positions of the adsorbed oxygen on the Cu_192_/MgO and Cu(7 × 5 × 5) NW models, with identical adsorption positions being shown in the same colors. To make sure that the results are comparable, the lattice constant of MgO was modified such that it fits the copper bulk lattice. For both models, we calculated adsorption energies for fixed (full circles) and fully relaxed Cu (open circles). For fully relaxed Cu, the oxide support was rotated by 45° so that the interface atoms of the copper nanowire face both magnesium and oxygen atoms of the support. The reason for this approach is that an unrotated nanowire, when fully relaxed, visibly distorts and bends to form patches, where Cu fits to the underlying MgO lattice, which we do not consider a realistic model of a Cu NP. As shown in Figure S2, the adhesion energy computed for a periodic interface rotated by 45° is very similar to that shown in Figure 2. For the models of fixed-geometry calculations, the MgO has not been rotated by 45°.

As can be seen from Figure 7, there are only small differences between the supported Cu_192_ and NW models when identical adsorption sites are compared. As established earlier, the only marked influence of the support on oxygen adsorption energies is given for adsorption at the Cu/MgO interface (see Figure 3). This adsorption is about ∼0.6 eV stronger, both for the interfaces of Cu_192_/MgO and Cu_NW_/MgO. We hence conclude that the NW model does reproduce the outcome of calculations with larger particles well.

## Conclusions

We have systematically investigated the extent of metal–support interactions using DFT calculations. Choosing MgO as a nonreducible support and copper nanoparticles of various sizes, we showed that the electronic effect of MgO on the reactivity of Cu, as measured by the oxygen adsorption energy, is rather small (about 0.1 eV), independent of particle size or shape. The only adsorption site where a strong influence of the support was present was the direct interface between MgO and the copper particles. When oxygen was bound to both MgO and Cu, an increased adsorption energy was obtained. Due to the lack of geometry optimizations, our models were not able to reproduce the interface site accurately. We further introduced a nanowire model, inspired by similar recent investigations in the literature, and showed that it is indeed representative of supported copper nanoparticles. Due to its small size, this model is able to predict interface sites efficiently while being representative of large nanoparticles.

**Figure 8:**
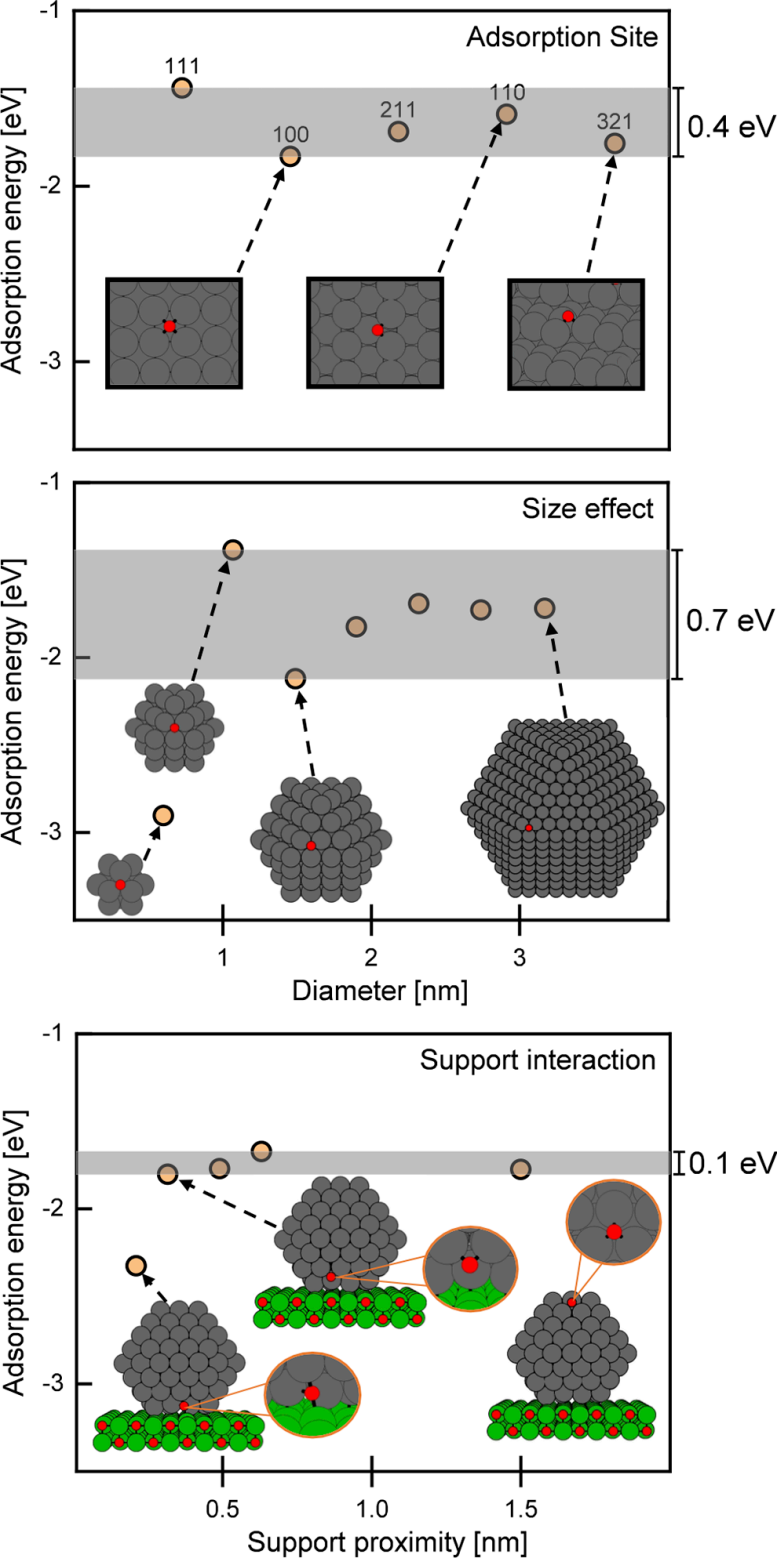
Above: Oxygen adsorption energy calculated on different copper surfaces ({111}, {100}, {211}, {110}, and {321}) indicating the influence of different adsorption sites. Middle: Oxygen chemisorption energy calculated on the free-standing fixed geometries of cuboctahedral copper structures (from 13 to 1415 atoms) revealing the influence of the particle size on oxygen adsorption. Bottom: Oxygen adsorption energies calculated on a MgO-supported Cu_192_ particle against the distance of the adsorbate from the oxide interface (in nm). The shaded areas as well as the scale bars (with values) on the right side of the plots represent the difference between the highest and lowest calculated oxygen adsorption energies. All energies are given in eV.

A schematic comparison of the influence of the metal–support interaction with those stemming from particle size effects and particle faceting is shown in Figure 8. Differences in binding energies from various adsorption sites on different facets (from Cu(111), CN = 9 to Cu(321), CN = 6) are on the order of 0.4 eV. The particle size effect is slightly larger (about 0.7 eV for particles > 1 nm) but vanishes at diameters above approximately 2.5 nm (an oxygen binding energy difference of around 0.1 eV between the particles above 2 nm). The influence of the MgO support results in moderate deviations of about 0.1 eV. Note that we observe remarkable differences for two cases, which are (1) extremely small clusters (Cu_13_ (∼0.5 nm) binding oxygen more strongly by ∼1 eV) and (2) the exact interface of the MgO support with the copper particle (∼0.5 eV) where oxygen binds to both MgO and Cu. We note that MgO is a nonreducible support, and the conclusions drawn here are not directly transferable to reducible supports such as, for example, ceria. In these cases, we would expect additional effects, such as the formation of oxygen vacancies, to play an important role, both for the electronic interaction between the support and metal particle but also for the reaction mechanism occurring at the interface. Extending our computational models to accurately describe these types of supports will be the subject of future research directions.

Using the Cu/MgO system, we have investigated the impact of metal–support interactions for a nonreducible oxide on the reactivity of copper, in addition to effects from particle size and surface faceting. While the Cu/MgO system is interacting strongly (−0.5 to −0.2 eV per copper surface atom depending on the specific structure), the support is nonreducible. The effect of reducible oxidic supports such as, e.g., CeO_2_ or ZnO might be substantially larger as electron donation could play a larger role.

## Supporting Information Available

Studied structures in Cartesian coordinates (ZIP)
Cohesive energies of the free-standing and MgO-supported copper nanoparticles; adhesion energies of copper nanoparticles on MgO; distance between the copper nanoparticles on MgO; influence of MgO support on oxygen binding energy; Cartesian coordinates of structures (PDF)
