# Supplementary material for: Metal–Support Interactions in Heterogeneous Catalysis: DFT Calculations on the Interaction of Copper Nanoparticles with Magnesium Oxide
Source: ACS Omega. 2023 Mar 7;8(11):10591–9. doi: 10.1021/acsomega.3c00502 (PMC10034847; doi:10.1021/acsomega.3c00502)
Supplement: Supplementary file 2 — ao3c00502_si_002.pdf [file ao3c00502_si_002.pdf]

Metal-Support Interactions in Heterogeneous Catalysis: DFT Calculations on the Interaction of Copper Nanoparticles with Magnesium Oxide - Supporting Information

Amir H. Hakimioun,† Bart D. Vandegehuchte,§ Daniel Curulla-Ferre,§ Kamila Kaźmierczak,§ Philipp N. Plessow,† and Felix Studt†,‡,*

†Institute of Catalysis Research and Technology, Karlsruhe Institute of Technology, Hermann-von-Helmholtz Platz 1, 76344 Eggenstein-Leopoldshafen, Germany

‡Institute for Chemical Technology and Polymer Chemistry, Karlsruhe Institute of Technology, Engesserstrasse 18, 76131 Karlsruhe, Germany

§TotalEnergies One Tech Belgium, B-7181 Seneffe, Belgium

*Corresponding author: felix.studt@kit.edu

TABLE OF CONTENTS

S1. Cohesive energies of the free-standing and MgO-supported copper nanoparticles

S2 Adhesion energies of copper nanoparticles on MgO

S3. Distance between the copper nanoparticles on MgO

S4. Influence of MgO support on oxygen binding energy

S5. Cartesian coordinates of structures

S1. Cohesive energies of the free-standing and MgO-supported copper nanoparticles

The stabilization effect of the magnesium oxide support on the copper nanoparticles is shown in **Figure S1**. A margin stabilizing effect of the support on the particles is observable. The cohesive energies were calculated using the following equation:

$$E_{Cohesive} = \frac{E_{Cu/MgO} - E_{MgO} - (N \times E_{Cu\ atom})}{N}$$

where $E_{Cu/MgO}$ is the total energy of the MgO supported copper nanoparticle, E_{MgO} is the energy of MgO, N is the number of copper atoms in the system and $E_{Cu\ atom}$ stands for the energy of a single copper atom (2.37 eV). The dashed line in the figure represents the cohesive energy of copper bulk on MgO (the structure is described in the main text of the paper).

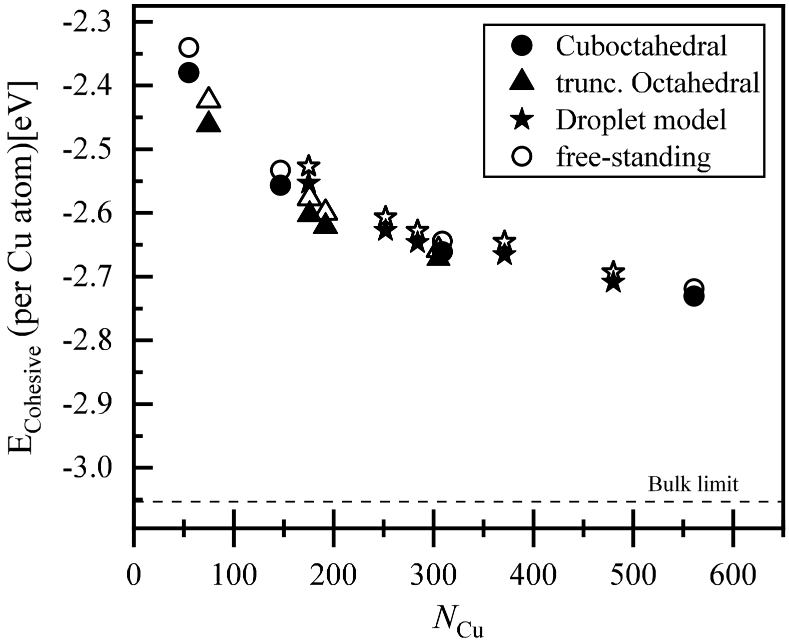

Figure S1. Calculated cohesive energies (per number of copper surface atoms facing the oxide support) both for MgO-supported and unsupported fixed-geometry copper nanoparticles versus the number of copper atoms in particles. The solid circle, triangle and star shapes represent cuboctahedral, truncated octahedral and droplet model geometries of the studied nanoparticles, respectively. The hollow shapes indicate the unsupported particles.

S2. Adhesion energies of copper nanoparticles on MgO

The calculated adhesion energies of copper nanoparticles on MgO are shown in **Figure S2(a)**. The represented energies are relative to the number of copper nanoparticle's interface atoms facing MgO(100) surface with their {100}(circle shapes) or {111} (triangle shapes) facets.

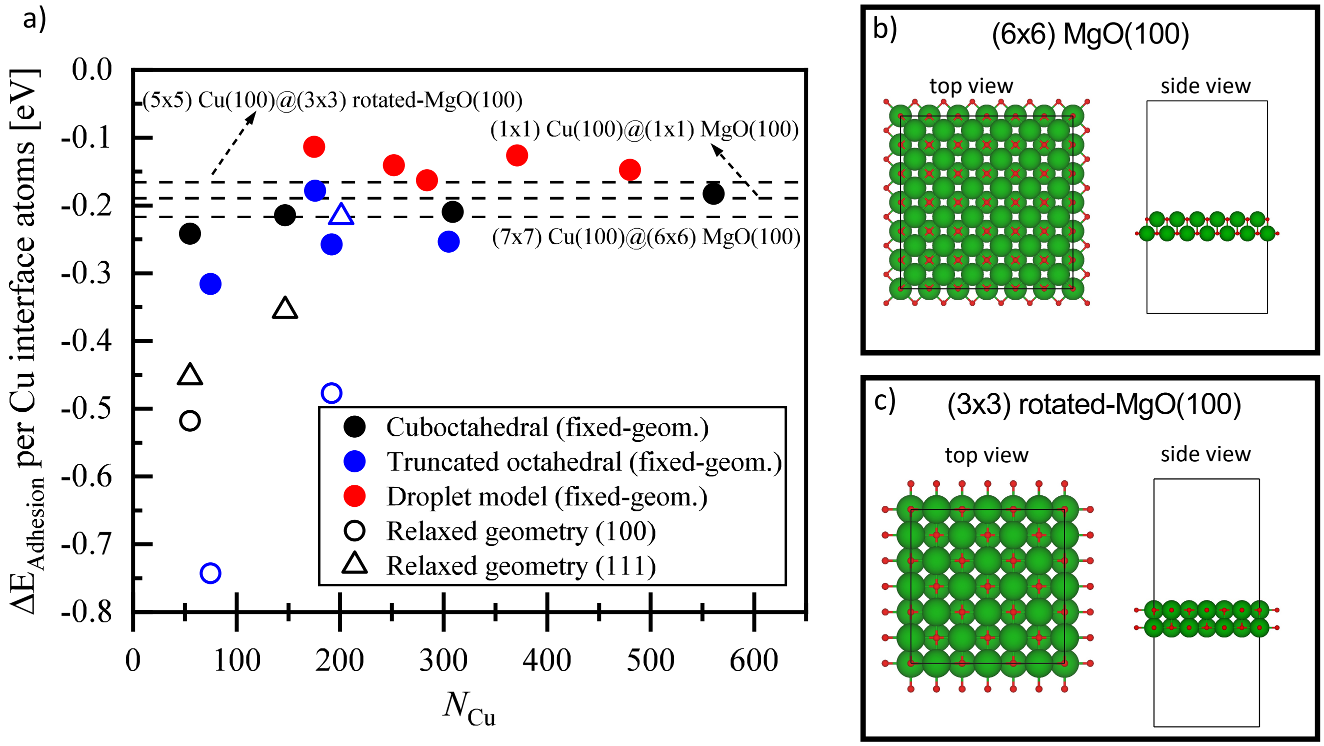

Figure S2. (a) Calculated adhesion energies of copper NPs supported by MgO per interface surface copper atoms against the total number of Cu atoms in the nanoparticles. Solid and hollow shapes represent the fixed-geometries and fully relaxed Cu NPs on MgO, respectively. Circle shapes are used to show the adhesion energies of copper NPs on the MgO via their Cu(100) facets and the triangle shapes are used for the adhesion of Cu NPs on MgO surface via their Cu(111) facets. Black, blue and red colors were used to differentiate the series of cuboctahedral (Cu55, Cu147, Cu309, and Cu561), truncated octa-hedral (Cu75, Cu176, Cu192, and Cu305) and droplet-like (Cu175, Cu252, Cu284, Cu371, and Cu480) nanoparticles, respectively. **(b)** and **(c)** represent top and side views of (6x6) MgO(100) and (3x3) 45° rotated-MgO(100) models, respectively.

S3. Distance between the copper nanoparticles on MgO

In order to investigate the influence of the distance between the periodic images of Cu/MgO systems, the oxygen adsorption energy on different positions (varying the distance of the oxygen atom from the support) on the copper nanoparticle with truncated octahedral geometries (Cu_{176}, Cu_{192} and Cu_{305}) with different distances between the periodic images of the structures was calculated. The oxygen adsorption energies on each geometrical series are referenced to the values obtained from the calculations on the structures with the longest distance between the periodic images. The dashed lines with the titles indicate the size of the largest MgO slabs (in *x*- and *y*-directions) used as references.

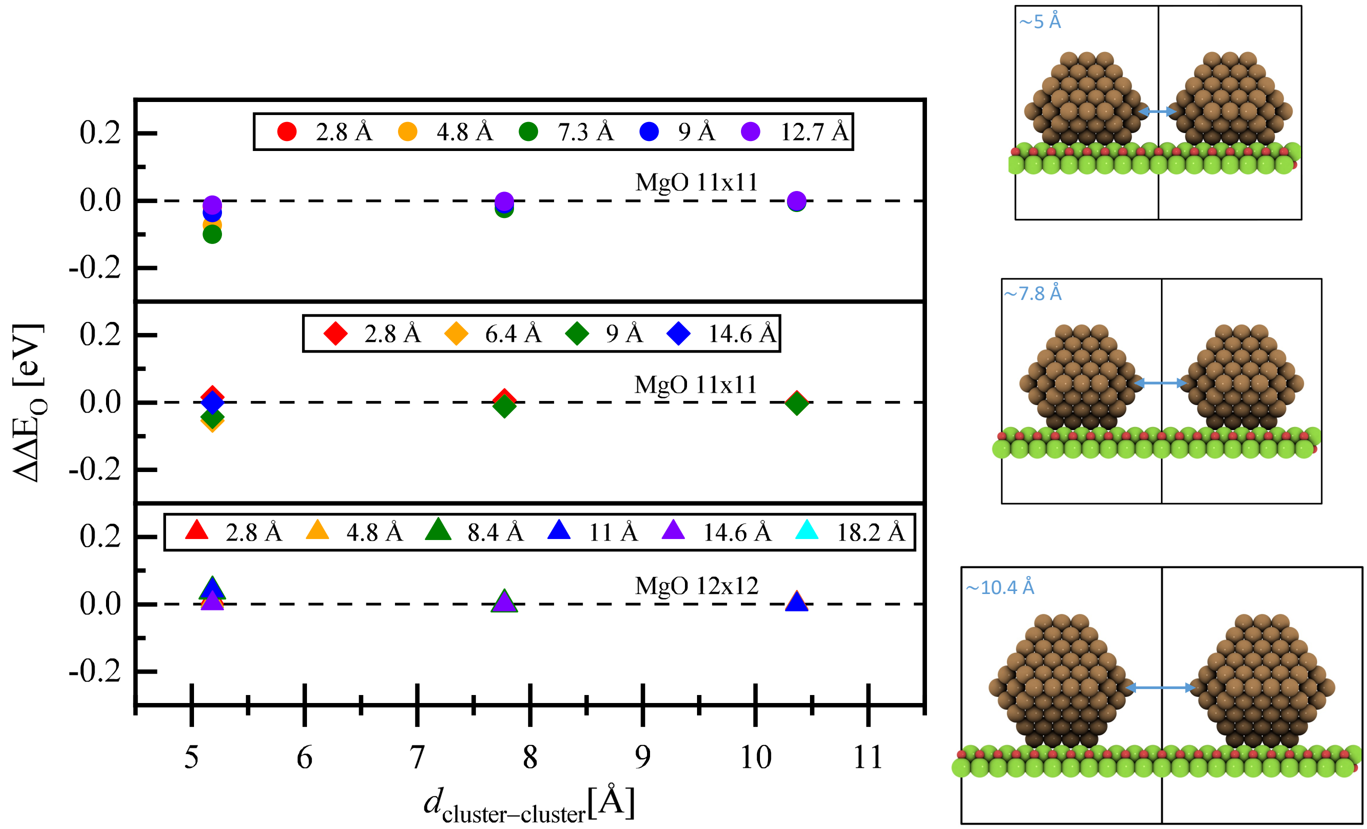

Figure S3. Left: The calculated oxygen adsorption energies difference on fixed-geometries of Cu_{176} (top figure), Cu_{192} (middle figure) and Cu_{305} (bottom figure) nanoparticles with respect to the distance between the copper nanoparticles and their periodic images. The values shown in legends represent the distance of oxygen atom, adsorbed on particles, from the oxide surface in angstrom. All the oxygen chemisorption energies are referenced to the values obtained from the calculate values of the same systems on longest distance between the particles. The dashed lines represent the referenced value calculated for oxygen adsorption energy on copper nanoparticles supported on the specified size of the magnesium oxide (number of magnesium atoms in *x*- and *y*-directions). **Right:** from top to bottom the repeated (in *x*-direction) structures of Cu_{176}, Cu_{192} and Cu_{305} supported on different sizes of MgO support. The distances between the particles with their periodic images are shown in figures (in blue color).

S4. Influence of MgO support on oxygen binding energy

The slight influence of MgO support on the properties of the copper nanoparticles is depicted in **Figure S4**. The mentioned influence is observable as the difference of oxygen adsorption energy, on different positions, calculated on three big nanoparticles with different geometries (Cu_{305}, Cu_{480}, and Cu_{561}), by the presence or absence of MgO support (solid circles and hollow circles, respectively). The GCN descriptor was used to show the divergence of the adsorption positions on NPs. In general, the highest effect of the MgO support on the oxygen adsorption energy on Cu NPs of 0.16 eV was predicted among different calculated systems.

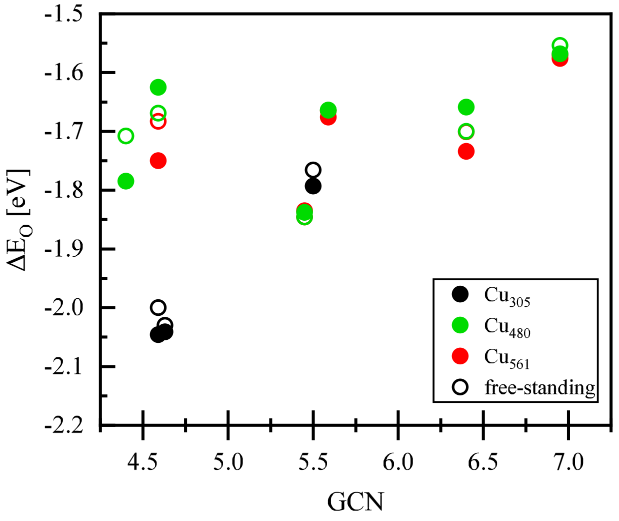

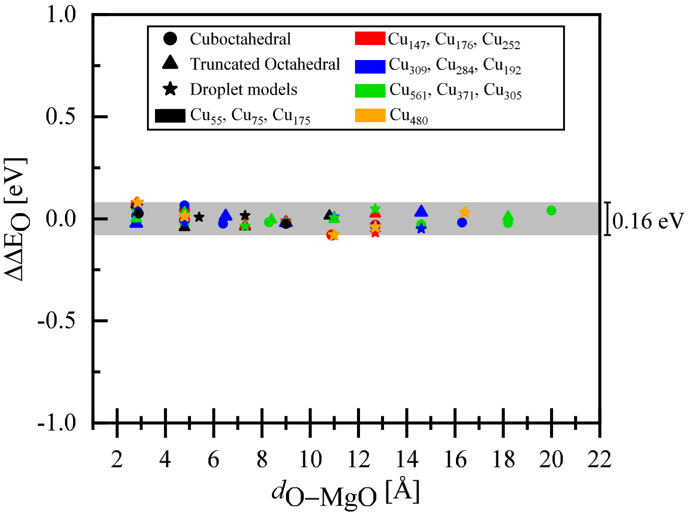

Figure S4: Left: Oxygen adsorption energy calculated on both MgO supported and unsupported fixed-geometries of copper nanoparticles with 305, 480 and 561 atoms against the generalized coordination number (GCN) of the adsorption positions. The adsorption positions were modelled identical for the systems with presence of the support and without it. The solid circle shapes represent the supported Cu_{305} (in black), Cu_{480} (in green) and Cu_{561} (in red) and the hollow shapes show the same calculations on the unsupported nanoparticles. **Right:** The oxygen chemisorption energy difference between the calculated values and the referenced values obtained from the zero slope fitted line of the points of each series, shown in Figure 4 of the main text, with respect to the distance of the adsorbate (oxygen atom) from the support in angstrom. All the energies shown are in eV.
